# Outcomes of ceftriaxone 2 g versus 1 g daily in hospitalized patients with pneumonia: a nationwide retrospective cohort study

**DOI:** 10.1093/jac/dkaf189

**Published:** 2025-06-10

**Authors:** Jumpei Taniguchi, Shotaro Aso, Hiroki Matsui, Kiyohide Fushimi, Hideo Yasunaga

**Affiliations:** Department of Clinical Epidemiology and Health Economics, School of Public Health, The University of Tokyo, Tokyo, Japan; Department of Health Services Research, Graduate School of Medicine, The University of Tokyo, Tokyo, Japan; Department of Clinical Epidemiology and Health Economics, School of Public Health, The University of Tokyo, Tokyo, Japan; Department of Health Policy and Informatics, Graduate School of Medical and Dental Sciences, Institute of Science Tokyo, Tokyo, Japan; Department of Clinical Epidemiology and Health Economics, School of Public Health, The University of Tokyo, Tokyo, Japan

## Abstract

**Objectives:**

Ceftriaxone is widely used for hospitalized patients with community-acquired pneumonia, but its optimal dosage remains unclear.

**Methods:**

We retrospectively identified patients diagnosed with pneumonia between July 2010 and March 2022 from the Diagnosis Procedure Combination inpatient database in Japan. They were categorized into those receiving 2 or 1 g/day of ceftriaxone within the first 2 days of hospitalization. The primary outcome was 30-day in-hospital mortality. The secondary outcomes included overall adverse events (composite of biliary tract infection, *Clostridioides difficile* infection and allergic reactions) and each adverse event. A subgroup analysis was conducted for patients requiring mechanical ventilation. Propensity-score overlap-weighting analysis was used for comparisons.

**Results:**

Among the 471 694 eligible patients, 63.3% received 2 g/day and 36.7% received 1 g/day of ceftriaxone. Propensity-score analysis showed no significant difference in 30-day in-hospital mortality between the two groups [4.5% versus 4.6%; risk difference (RD), −0.1%; 95% confidence interval (CI), −0.3% to 0.1%; *P* = 0.219]. Overall adverse events were slightly higher in the 2 g/day group (1.9% versus 1.8%; RD, 0.1%; 95% CI, 0.0%–0.2%; *P* = 0.007), particularly the proportion of *C. difficile* infection. In the subgroup analysis of patients requiring mechanical ventilation, the 2 g/day regimen was associated with lower 30-day mortality (17.2% versus 20.4%; RD, −3.2%; 95% CI, −5.6% to −0.9%; *P* = 0.006).

**Conclusions:**

While a ceftriaxone dose exceeding 1 g/day may not be necessary for routine pneumonia treatment, a 2 g/day regimen may be considered for patients with severe pneumonia requiring mechanical ventilation.

## Introduction

Community-acquired pneumonia remains one of the leading causes of morbidity and mortality worldwide, representing a significant burden on healthcare systems.^1,2^ Effective management of pneumonia often involves the use of empiric antibiotic therapy to cover a range of potential pathogens, with ceftriaxone being one of the most commonly prescribed antibiotics.^3^ According to the international guidelines of the American Thoracic Society and Infectious Diseases Society of America, ceftriaxone in a dose of 1–2 g daily is recommended for hospitalized patients with community-acquired pneumonia. However, there is no clear consensus on the optimal dosing regimen.

Previous studies have reported contradictory findings regarding the efficacy of different ceftriaxone dosing strategies in community-acquired pneumonia.^4–8^ A small randomized controlled trial, retrospective studies and systematic review and meta-analysis have suggested that both 1 and 2 g/day regimens have comparable clinical effectiveness.^4–6,8^ Conversely, studies focusing on critically ill patients with community-acquired infection requiring intensive care admission indicated the potential benefits of 2 g/day ceftriaxone regimen, with some experts advocating for higher doses in severe cases.^7^ While managing community-acquired pneumonia, we need to consider the regional antibiogram patterns and demographic variations among patients. A retrospective cohort study conducted in Japan examined the non-inferiority of 1 g/day ceftriaxone dosing compared to 2 g/day ceftriaxone dosing; however, certain limitations, such as a small sample size were noted, highlighting the need for more robust and generalizable data.^5^ Additionally, the impact of ceftriaxone dosage on the incidence of adverse events, including ceftriaxone-specific complications such as biliary infections mediated by pseudolithiasis and *Clostridioides difficile* infection, has been poorly investigated.^4,8^

This study aimed to evaluate the outcomes and adverse events of ceftriaxone regimens of 1 and 2 g/day in hospitalized patients with pneumonia using a Japanese national inpatient database.

## Methods

### Study design and setting

This retrospective cohort study utilized the Japanese Diagnosis Procedure Combination inpatient database, which includes comprehensive data from over 1200 hospitals across Japan.

### Data source

Data were obtained from the Diagnosis Procedure Combination database in Japan.^9^ This is an inpatient database that serves as a comprehensive repository of clinical and administrative data. It includes patient demographics such as age, sex, height and weight, along with the primary diagnoses, pre-existing comorbidities at admission and complications arising during hospitalization. Diagnoses, comorbidities and complications were coded using the International Classification of Diseases, 10th Revision (ICD-10). Additional details extracted from the database included admission and discharge dates, prescribed medications, surgical procedures and hospital identifiers. With data collected from over 1200 hospitals across Japan, this database has become a pivotal resource for healthcare research.^10^ A validation study has demonstrated the database’s accuracy, reporting diagnostic specificity above 96%, sensitivity between 50% and 80% and both sensitivity and specificity for surgical and procedural records exceeding 90%.^11^

### Patient selection

We identified patients who were diagnosed with pneumonia (ICD-10 codes: J10.x–J18.x, J69.x) between July 2010 and March 2022. Only the first hospitalization was considered if the patients were hospitalized more than once for the diagnosis of pneumonia during the study period. We included patients who received ceftriaxone within 2 days of admission. We excluded patients who received combination therapy with antibiotics other than macrolides or tetracyclines within 2 days of admission; patients who were treated with antifungal or antivirus drugs within 2 days of admission; patients who were initially treated with ceftriaxone at doses other than 1 or 2 g/day; patients who died or were discharged within 2 days of admission; patients with pyothorax (J86.x) or lung abscess (J85.0–J85.2); patients who underwent chest tube insertion within 2 days of admission; patients aged <15 years; and pregnant patients.

### Exposure

Patients were divided into those who received ceftriaxone at 2 g/day (2 g/day group) and those who received ceftriaxone at 1 g/day (1 g/day group) within 2 days of admission.

### Covariates

Covariates were classified into three main categories: patient characteristics at admission, nutritional intake and treatments within the first 2 days of hospitalization and hospital characteristics. Patient characteristics included variables such as age, sex, body weight at admission, smoking status (categorized as non-smoker, current smoker, or former smoker), Glasgow Coma Scale score at admission, Barthel Index,^12^ Charlson Comorbidity Index,^13^ pneumonia severity score, admission to intensive care or high care units, arrival by ambulance, fiscal year of admission, diagnosis of aspiration pneumonia and comorbidities. Comorbidities assessed included hypertension, diabetes mellitus, dyslipidaemia, lung diseases (chronic obstructive pulmonary disease, interstitial pneumonia, bronchiectasis, nontuberculous mycobacterial infections, fungal lung disease, lung cancer and chronic respiratory failure), oesophageal disorders and dysphagia, cerebrovascular diseases, neurological disorders, cardiovascular diseases, liver diseases, gallbladder disease, biliary tract disease, chronic kidney failure, haematologic malignancies, non-haematologic malignancies and dementia (Table S1, available as Supplementary data at *JAC* Online). Nutritional intake was defined as the mode of nutritional support administered within the first 2 days of admission, and was categorized as oral feeding, tube feeding, or total parenteral nutrition. Treatments within 2 days of admission included macrolides, tetracyclines, oxygen therapy, mechanical ventilation, vasopressor use, renal replacement therapy and administration of specific medications such as corticosteroids, proton pump inhibitors, hypnotics and antipsychotics. Hospital characteristics were defined as whether the hospital was a teaching or non-teaching hospital.

The Barthel Index was grouped into four categories: 0, 5–50, 55–95 and 100. Pneumonia severity score in this study was assessed using the A-DROP scoring system. The A-DROP scoring system, developed by the Japanese Respiratory Society, is an adaptation of the CURB-65 score for assessing the severity of community-acquired pneumonia.^14,15^ The A-DROP scoring system includes five criteria, each represented by a corresponding letter as well as CURB-65: age (≥70 years for men or ≥75 years for women), dehydration (blood urea nitrogen ≥ 21 mg/dL or clinical signs), respiratory status (SpO₂ ≤ 90% or PaO₂ ≤ 60 torr), orientation disturbance and pressure (systolic blood pressure ≤ 90 mmHg). Each criterion scores one point, with higher scores indicating more severe pneumonia. This system is tailored to Japanese clinical settings and complements the CURB-65 model.^16^ Steroid use was defined as the administration of corticosteroids equivalent to at least 200 mg/day of hydrocortisone.

### Outcomes

The primary outcome was 30-day in-hospital mortality. The secondary outcomes were overall adverse events and components of the overall adverse events. Overall adverse events were defined as a composite outcome of biliary complications, *C. difficile* infection and allergic reactions occurring during hospitalization. Biliary complications included cholecystitis or cholangitis during hospitalization (K80.0, K80.1, K80.3, K80.4, K81.x and K83.0) and percutaneous, endoscopic, or surgical interventions performed on the gallbladder or biliary tract. *C. difficile* infection was defined as a recorded diagnosis of *C. difficile* infection (A04.7) or prescription of antibiotics commonly used for *C. difficile* treatment (oral vancomycin, oral metronidazole, or fidaxomicin). Patients with a recorded diagnosis of enterocolitis caused by methicillin-resistant *Staphylococcus aureus* (A04.8) were excluded from the analysis of *C. difficile* infection.^17^ Allergic reactions were identified as complications of allergy-related disease occurring during hospitalization (L23.9, L27.x, L50.x–L51.x, L53.x, T78.2 and T78.4).

### Statistical analysis

We used a propensity-score overlap-weighting approach to compare the outcomes between the two groups.^18,19^ Propensity scores for ceftriaxone 2 g/day use were estimated using a logistic regression model, with ceftriaxone 2 g/day as the dependent variable and all covariates as the independent variables. In the overlap-weighting method, weights are assigned to each patient based on their probability of receiving the alternate treatment.^19^ Specifically, patients receiving 2 g/day of ceftriaxone were weighted by their likelihood of not receiving it (1—propensity score), while those receiving 1 g/day were weighted by their likelihood of receiving it (propensity score). This method balances covariates between the groups by assigning greater weights to patients with a substantial probability of receiving either treatment, thereby focusing the analysis on individuals in the region of common support and reducing model dependence, while retaining many patients in the analysis. To evaluate balance, standardized differences were calculated for each covariate, with absolute values below 10% indicating acceptable balance.^20,21^

### Subgroup analysis and sensitivity analysis

We performed subgroup analyses based on the receipt of mechanical ventilation, bedridden status (Barthel Index = 0 or Barthel Index > 0), diagnosis of aspiration pneumonia, underweight (body mass index < 18.5 kg/m^2^ or ≥18.5 kg/m^2^) and receipt of renal replacement therapy. The subgroup analyses based on the receipt of mechanical ventilation and bedridden status were performed to assess the effect of 2 g/day of ceftriaxone in critically ill patients and frail individuals, respectively.

We conducted several sensitivity analyses. This included exclusion of patients who received combination therapy with macrolides or tetracyclines to account for the potential confounding effects of these antibiotics. We performed univariate and multivariate logistic regression analyses to assess the association between ceftriaxone dosage and 30-day in-hospital mortality, thereby evaluating the impact of potential confounding.

Continuous variables were analysed with *t*-tests and reported as means and standard deviations. Categorical variables were evaluated using the chi-square test and presented as counts and percentages. A two-sided *P*-value less than 0.05 was considered indicative of statistical significance. All statistical procedures were conducted using STATA/SE version 18.0 (StataCorp, College Station, TX, USA).

This study was approved by the Institutional Review Board of the University of Tokyo (3501-5, 19 May 2021). The board waived the need for informed consent as this was a retrospective study.

## Results

**Figure 1.**
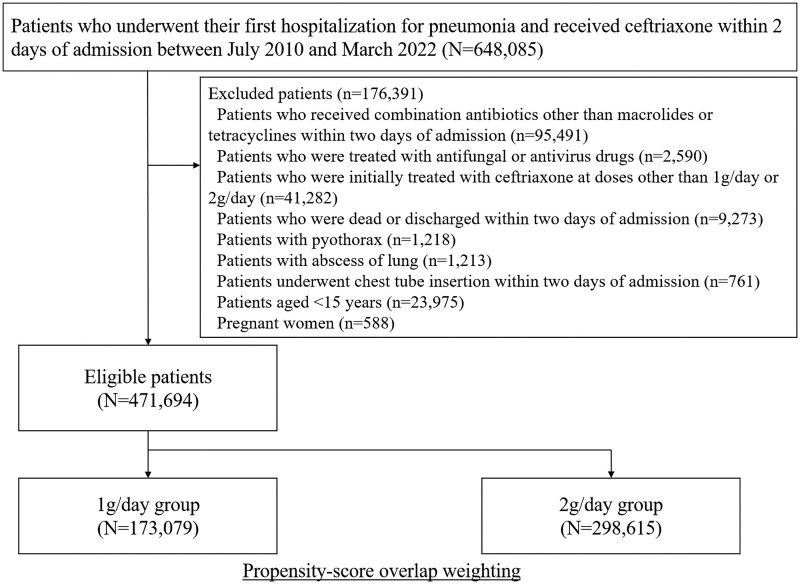
Study design.

We identified 648 085 patients who were admitted for pneumonia and received ceftriaxone between July 2010 and March 2022 (Figure 1). A total of 471 694 patients were found to be eligible. Among them, 298 615 (63.3%) patients received 2 g/day of ceftriaxone and 173 079 (36.7%) received 1 g/day of ceftriaxone. The mean treatment duration was 7.3 days (standard deviation, 3.5) in the 2 g/day group and 7.4 days (standard deviation, 3.7) in the 1 g/day group.

Table 1 shows the baseline characteristics of the study groups before and after propensity-score overlap weighting. Patients in the 2 g/day group were younger, had higher Barthel Index scores and lower pneumonia severity scores. As the fiscal year became more recent, the proportion of patients treated with the 2 g/day regimen tended to increase. The proportion of patients with aspiration pneumonia in the 2 g/day group was lower than that in the 1 g/day group. The proportion of patients taking oral nutrition, using macrolides as combination therapy, and getting admission to teaching hospitals was higher in the 2 g/day group than in the 1 g/day group. In the unadjusted analysis, 30-day in-hospital mortality in the 2 g/day group was lower than that in the 1 g/day group [5.8% versus 6.7%; risk difference (RD), −0.8%; 95% confidence interval (CI), −1.0% to −0.7%; *P* < 0.001]. The proportion of overall adverse events in the 2 g/day group was lower than that in the 1 g/day group (2.2% versus 2.4%; RD, −0.1%; 95% CI, −0.2% to −0.0%; *P* = 0.004).

**Table 1.** Patient characteristics

Variables	Unweighted			Propensity-score overlap weighted		
	1 g/day (*n* = 173 079)	2 g/day (*n* = 298 615)	Standardized difference	1 g/day (*n* = 131 582)	2 g/day (*n* = 131 582)	Standardized difference
Age, years, mean (SD)	79.5 (14.5)	76.8 (15.5)	−17.9	76.3 (16.5)	76.3 (15.6)	0.0
Male, *n* (%)	91 638 (52.9%)	170 452 (57.1%)	8.4	70 986 (53.9%)	70 986 (53.9%)	0.0
Body weight, kg, mean (SD)	45.5 (21.4)	47.6 (20.4)	10.6	52.1 (17.7)	52.1 (13.8)	0.0
Smoking history, *n* (%)						
Non-smoker	109 616 (63.3%)	175 721 (58.8%)	−9.2	88 867 (67.5%)	88 867 (67.5%)	0.0
Current/past smoker	42 487 (24.5%)	87 486 (29.3%)	10.9	42 715 (32.5%)	42 715 (32.5%)	0.0
Missing data	20 976 (12.1%)	35 408 (11.9%)	−1.4			
GCS on admission, mean (SD)	14.4 (1.6)	14.5 (1.5)	5.9	14.6 (1.3)	14.6 (1.3)	0.0
Barthel Index on admission, *n* (%)						
0	48 058 (27.8%)	69 314 (23.2%)	−11.1	27 745 (21.1%)	27 745 (21.1%)	0.0
5–50	37 007 (21.4%)	56 341 (18.9%)	−6.9	30 006 (22.8%)	30 006 (22.8%)	0.0
55–95	23 431 (13.5%)	43 478 (14.6%)	2.3	25 008 (19.0%)	25 008 (19.0%)	0.0
100	40 991 (23.7%)	89 946 (30.1%)	14.7	48 823 (37.1%)	48 823 (37.1%)	0.0
Missing data	23 592 (13.6%)	39 536 (13.2%)	−1.6			
Charlson comorbidity index, mean (SD)	1.4 (1.5)	1.3 (1.5)	−6.5	1.3 (1.4)	1.3 (1.4)	0.0
Pneumonia severity score, mean (SD)	1.7 (1.1)	1.6 (1.1)	−10.4	1.6 (1.1)	1.6 (1.1)	0.0
ICU admission, *n* (%)	1061 (0.6%)	2210 (0.7%)	1.6	712 (0.5%)	712 (0.5%)	0.0
HCU admission, *n* (%)	3033 (1.8%)	6688 (2.2%)	3.0	2135 (1.6%)	2135 (1.6%)	0.0
Ambulance transport, *n* (%)	63 051 (36.4%)	110 364 (37.0%)	0.2	40 145 (30.5%)	40 145 (30.5%)	0.0
Fiscal year, *n* (%)						
2010–11	24 552 (14.2%)	27 283 (9.1%)	−18.6	17 343 (13.2%)	17 343 (13.2%)	0.0
2012–13	28 266 (16.3%)	34 998 (11.7%)	−12.8	18 335 (13.9%)	18 335 (13.9%)	0.0
2014–15	34 203 (19.8%)	49 022 (16.4%)	−7.4	24 358 (18.5%)	24 358 (18.5%)	0.0
2016–17	34 203 (19.8%)	63 177 (21.2%)	4.6	28 258 (21.5%)	28 258 (21.5%)	0.0
2018–19	32 448 (18.7%)	74 690 (25.0%)	16.4	28 579 (21.7%)	28 579 (21.7%)	0.0
2020–21	19 407 (11.2%)	49 445 (16.6%)	14.9	14 709 (11.2%)	14 709 (11.2%)	0.0
Aspiration pneumonia, *n* (%)	43 839 (25.3%)	61 325 (20.5%)	−10.4	8692 (6.6%)	8692 (6.6%)	0.0
Hypertension, *n* (%)	50 449 (29.1%)	84 050 (28.1%)	−2.8	38 726 (29.4%)	38 726 (29.4%)	0.0
Diabetes mellitus, *n* (%)	31 803 (18.4%)	55 402 (18.6%)	0.2	25 106 (19.1%)	25 106 (19.1%)	0.0
Dyslipidaemia, *n* (%)	13 829 (8.0%)	26 345 (8.8%)	2.9	12 286 (9.3%)	12 286 (9.3%)	0.0
Lung disease, *n* (%)						
Chronic obstructive pulmonary disease	12 915 (7.5%)	27 578 (9.2%)	6.5	11 781 (9.0%)	11 781 (9.0%)	0.0
Interstitial Pneumonia	4028 (2.3%)	9548 (3.2%)	4.9	3399 (2.6%)	3399 (2.6%)	0.0
Bronchiectasis and NTM of the lungs	1995 (1.2%)	3914 (1.3%)	1.1	1965 (1.5%)	1965 (1.5%)	0.0
Fungal lung disease	297 (0.2%)	693 (0.2%)	1.2	221 (0.2%)	221 (0.2%)	0.0
Lung cancer	4093 (2.4%)	8546 (2.9%)	3.2	3404 (2.6%)	3404 (2.6%)	0.0
Chronic respiratory failure	3428 (2.0%)	6188 (2.1%)	0.5	2743 (2.1%)	2743 (2.1%)	0.0
Oesophageal disorders and dysphagia, *n* (%)	7312 (4.2%)	10 867 (3.6%)	−3.1	3475 (2.6%)	3475 (2.6%)	0.0
Cerebrovascular disease, *n* (%)	23 457 (13.6%)	34 699 (11.6%)	−5.9	13 610 (10.3%)	13 610 (10.3%)	0.0
Neurologic disease, *n* (%)	4761 (2.8%)	7349 (2.5%)	−2.1	2400 (1.8%)	2400 (1.8%)	0.0
Cardiovascular disease, *n* (%)	35 963 (20.8%)	55 358 (18.5%)	−6.3	25 875 (19.7%)	25 875 (19.7%)	0.0
Liver disease, *n* (%)	5493 (3.2%)	9257 (3.1%)	−0.4	4603 (3.5%)	4603 (3.5%)	0.0
Gallbladder disease, *n* (%)	1656 (1.0%)	2555 (0.9%)	−0.8	1044 (0.8%)	1044 (0.8%)	0.0
Biliary tract disease, *n* (%)	627 (0.4%)	982 (0.3%)	−0.3	415 (0.3%)	415 (0.3%)	0.0
Chronic kidney failure, *n* (%)	16 463 (9.5%)	18 805 (6.3%)	−11.3	10 350 (7.9%)	10 350 (7.9%)	0.0
Haematological malignancy, *n* (%)	1503 (0.9%)	3060 (1.0%)	2.0	1247 (0.9%)	1247 (0.9%)	0.0
Non-haematological malignancy, *n* (%)	11 782 (6.8%)	20 276 (6.8%)	0.0	8635 (6.6%)	8635 (6.6%)	0.0
Dementia, *n* (%)	25 356 (14.6%)	37 043 (12.4%)	−7.1	13 856 (10.5%)	13 856 (10.5%)	0.0
Nutrition within 2 days of admission day, *n* (%)						
Oral feeding	126 970 (73.4%)	233 608 (78.2%)	11.5	110 253 (83.8%)	110 253 (83.8%)	0.0
Tube feeding	1751 (1.0%)	2994 (1.0%)	−0.7	853 (0.6%)	853 (0.6%)	0.0
Total parenteral nutrition	811 (0.5%)	1125 (0.4%)	−1.2	423 (0.3%)	423 (0.3%)	0.0
Treatment within 2 days of admission, *n* (%)						
Oxygenation	88 945 (51.4%)	154 178 (51.6%)	0.1	61 722 (46.9%)	61 722 (46.9%)	0.0
Mechanical ventilation	2893 (1.7%)	6111 (2.0%)	2.6	2018 (1.5%)	2018 (1.5%)	0.0
Vasopressors	2494 (1.4%)	4574 (1.5%)	0.5	1425 (1.1%)	1425 (1.1%)	0.0
Renal replacement therapy	4128 (2.4%)	3722 (1.2%)	−7.5	2269 (1.7%)	2269 (1.7%)	0.0
Macrolides	17 824 (10.3%)	47 137 (15.8%)	16.6	19 883 (15.1%)	19 883 (15.1%)	0.0
Tetracyclines	4340 (2.5%)	8271 (2.8%)	1.2	4295 (3.3%)	4295 (3.3%)	0.0
Steroids	8274 (4.8%)	17 839 (6.0%)	5.3	7073 (5.4%)	7073 (5.4%)	0.0
Proton pump inhibitors	28 638 (16.5%)	56 800 (19.0%)	6.4	22 242 (16.9%)	22 242 (16.9%)	0.0
Hypnotics	17 039 (9.8%)	26 427 (8.8%)	−4.0	8963 (6.8%)	8963 (6.8%)	0.0
Antipsychotics	3545 (2.0%)	6172 (2.1%)	1.4	662 (0.5%)	662 (0.5%)	0.0
Teaching hospital admission, *n* (%)	130 695 (75.5%)	241 446 (80.9%)	12.8	101 444 (76.1%)	101 444 (76.1%)	0.0

The total number of aetiologies does not add up to 100% as more than one cause can be assigned to a single patient.

GCS, Glasgow Coma Scale; HCU, high care unit; ICU, intensive care unit; NTM, non-tuberculosis mycobacterium; SD, standard deviation.

### Main analysis

After propensity-score overlap weighting, all baseline characteristics were well-balanced between the groups (Table 1). In the propensity-score analysis, there was no significant difference in 30-day in-hospital mortality between the two groups (4.5% versus 4.6%; RD, −0.1%; 95% CI, −0.3% to 0.1%; *P* = 0.219). However, the percentage of overall adverse events was slightly higher in the 2 g/day group (1.9% versus 1.8%; RD, 0.1%; 95% CI, 0.0%–0.2%; *P* = 0.007), along with an increase in the proportion of *C. difficile* infection (1.2% versus 1.1%; RD, 0.1%; 95% CI, 0.0%–0.2%; *P* = 0.014) (Table 2).

**Table 2.** Comparison of outcomes between the propensity score weighted groups

	1 g/day	2 g/day	Risk difference	95% confidence interval	*P*
Primary outcome (%)					
30-Day in-hospital mortality	4.6% (6.7%)	4.5% (5.8%)	−0.1	−0.3 to 0.1	0.219
Secondary outcome (%)					
Overall adverse events	1.8% (2.4%)	1.9% (2.2%)	0.1	0.0 to 0.2	0.007
Biliary complications	0.2% (0.4%)	0.2% (0.3%)	0.0	−0.0 to 0.0	1.000
*Clostridioides difficile* infection	1.1% (1.5%)	1.2% (1.4%)	0.1	0.0 to 0.2	0.014
Allergic reactions	0.5% (0.5%)	0.6% (0.6%)	0.0	−0.0 to 0.1	0.094

Values in parentheses indicate the crude outcomes.

### Subgroup analysis

**Figure 2.**
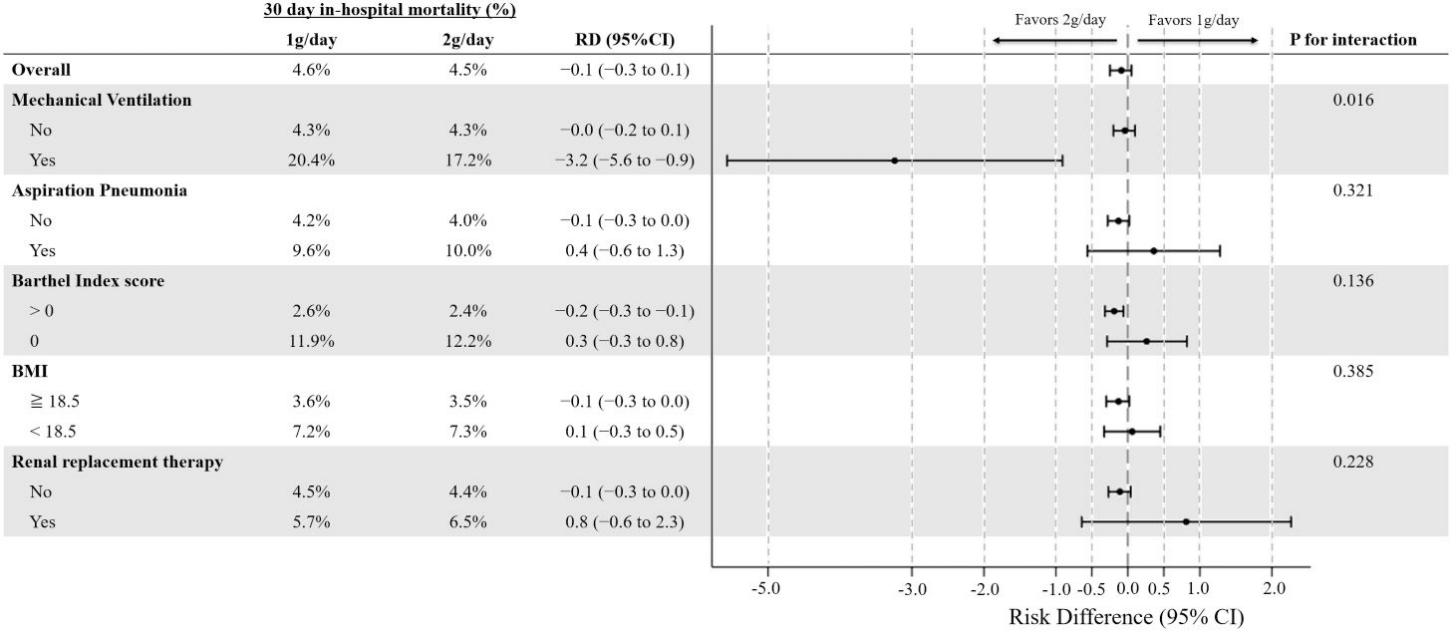
Subgroup analysis for 30-day in-hospital mortality between the propensity score weighted groups. BMI, body mass index; RD, risk difference; CI, confidence interval.

The results of the subgroup analysis are presented in Figure 2 and Figures S1–S4. Among patients with severe pneumonia who received mechanical ventilation within 2 days of admission, 30-day in-hospital mortality was lower in the 2 g/day group than in the 1 g/day group (17.2% versus 20.4%; RD, −3.2%; 95% CI, −5.6% to −0.9%; *P* = 0.006), while allergic reactions were slightly more frequent (0.6% versus 0.1%; RD, 0.5%; 95% CI, 0.1%–0.8%; *P* = 0.012) (Figure 2 and Figure S4). Among bedridden patients, the incidence of *C. difficile* infections was slightly higher in the 2 g/day group than in the 1 g/day group (2.7% versus 2.2%; RD, 0.4%; 95% CI, 0.2%–0.7%; *P* = 0.006) (Figure S3). Statistically significant interactions were observed for 30-day in-hospital mortality and allergic reactions among patients who received mechanical ventilation and for the incidence of *C. difficile* infection among bedridden patients (*P* for interaction < 0.05 for all; Figure 2, Figures S3 and S4). No significant differences were observed for other outcomes among patients with aspiration pneumonia, those with a body mass index < 18.5 kg/m^2^ and those who received renal replacement therapy (Figure 2 and Figures S1–S4).

The results of the sensitivity analysis were consistent with those of the main analysis (Tables S2–S4).

## Discussion

In this retrospective study, we compared the outcomes between 2 g/day versus a 1 g/day ceftriaxone regimen in patients with pneumonia. Overall, there was no significant difference in 30-day in-hospital mortality between the two dosing groups. However, among patients requiring mechanical ventilation, the 2 g/day regimen was associated with a significantly lower mortality rate. These findings suggest that while 2 g/day may not be necessary for routine pneumonia cases, it may offer a clinical benefit in patients with severe pneumonia requiring mechanical ventilation.

A major strength of our study is the use of a large, nationwide database to compare the treatment outcomes of different ceftriaxone dosing regimens, including adverse events, for the treatment of pneumonia. While a small randomized controlled trial comparing ceftriaxone dosages was conducted for community-acquired infections, no trial has specifically focused on patients with pneumonia.^4,6^ A systematic review and meta-analysis exists, but it indirectly compared the efficacy of different ceftriaxone dosing regimens.^4^ Although several retrospective studies have directly examined ceftriaxone dosing in pneumonia, these studies were limited by single-centre designs, small sample sizes and concerns regarding the statistical power and external validity.^5,8^ In contrast, our study utilized a large-scale database comprising data from over 1200 hospitals to directly examine the impact of ceftriaxone dosage on pneumonia outcomes. To the best of our knowledge, this is the largest study to compare the treatment outcomes of ceftriaxone dose regimens directly in patients with pneumonia.

The results of our study are consistent with those of previous studies that evaluated ceftriaxone dosage in patients with pneumonia.^4–6,8^ Pharmacokinetic studies of ceftriaxone in healthy adults have also shown that a 1 g/day regimen achieves sufficient serum concentrations above the minimum inhibitory concentrations for the major pneumonia pathogens, including *Streptococcus pneumoniae*, *Haemophilus influenzae* and *Moraxella catarrhalis*, and have demonstrated its antimicrobial activity.^22,23^ Our study found that a 2 g/day regimen may be potentially beneficial in patients with severe pneumonia requiring mechanical ventilation, consistent with the findings of previous studies on patients with severe community-acquired infections.^7^ Compared to healthy individuals, critically ill patients with normal renal function exhibit increased ceftriaxone clearance and volume of distribution.^7,24–26^ Therefore, our study results suggest that in patients with severe pneumonia, a 1 g/day ceftriaxone dose may not achieve sufficient plasma concentrations, and a 2 g/day ceftriaxone regimen should be considered for these patients.

Systematic reviews and meta-analyses examining the association between the antibiotic type and incidence of *C. difficile* infection have suggested that ceftriaxone carries a higher risk of *C. difficile* infection compared with other antibiotics commonly used for treating pneumonia.^27,28^ The increase in overall adverse events observed in our study, particularly the higher incidence of *C. difficile* infection among bedridden patients, is consistent with the result of a previous single-centre study comparing ceftriaxone 2 and 1 g/day regimens in patients with pneumonia.^8^ In patients with severe physical dependency and high risk of healthcare-associated infections, the baseline risk of *C. difficile* infection may already be high, and even a difference of approximately 1 g/day in ceftriaxone dosage can affect the incidence of *C. difficile* infections. Considering both the clinical effectiveness and adverse events, a regimen of 1 g/day of ceftriaxone may be sufficient for the treatment of mild to moderate pneumonia, especially in patients with a high risk of healthcare-associated infections, including severe physical dependency.

In the primary analysis, no dose-dependent increase in the incidence of allergic reactions was observed. However, among patients who underwent mechanical ventilation, the incidence was significantly higher in the 2 g/day ceftriaxone group. Although the exact cause remains unclear, cephalosporins are known to induce non-IgE-mediated allergic reactions.^29^ The difference may also be attributable to enhanced immune responses in critically ill patients, increased exposure to multiple medications, or more intensive clinical monitoring in this population.

This study has some limitations. First, we could not obtain clinical information regarding the diagnoses, such as the patients’ medical history, laboratory data, imaging findings and microbiological information. Although we included the medication use and procedures in the definition of diagnoses in addition to the recorded diagnoses during hospitalization, there may have been a potential misclassification bias. We defined *C. difficile* infection based on both the recorded diagnoses and use of relevant antibiotics. Since nucleic acid amplification testing was not widely available in Japan, patients with negative toxin test results but a high clinical suspicion of *C. difficile* infection were often empirically treated with antibiotics commonly used for its treatment.^30,31^ We excluded patients with a recorded diagnosis of methicillin-resistant *S. aureus* enterocolitis from the analysis of secondary outcomes. However, since microbiological test results were not available, further investigation is needed to evaluate the adverse events related to differences in ceftriaxone dosage.^17^ Second, the specific reasons for selecting either 2 or 1 g/day of ceftriaxone in this study were unclear. Although we adjusted for patient characteristics, including the pneumonia severity score A-DROP, the possibility of confounding by the indication influencing the results cannot be ruled out. Additionally, our results were derived from patients with pneumonia and may not be generalizable to other infections, such as urinary tract infections. Further research is needed to evaluate ceftriaxone dosing in patients with different clinical backgrounds. Third, due to the nature of the database, we could not determine whether the 2 g/day dose of ceftriaxone was administered as a single 2 g dose once daily or as 1 g twice daily. This distinction may influence pharmacokinetics and, consequently, clinical outcomes. A single-centre retrospective study from Japan compared once-daily (2 g once) and twice-daily (1 g twice) regimens in patients with community-acquired pneumonia and found no significant difference in 30-day mortality.^32^ However, further research is warranted to evaluate the clinical impact of different dosing strategies. Fourth, although this study utilized a large-scale database comprising data from over 1200 hospitals in Japan, the data were limited to a single country and predominantly derived from an Asian population. According to national antimicrobial resistance surveillance data, less than 5% of *S. pneumoniae* isolated from non-meningitis specimens in Japan have penicillin minimum inhibitory concentration (MIC) ≥ 4 mg/L, indicating a relatively low prevalence of resistance compared to other countries.^33^ Our findings are more applicable to regions where resistance rates of pathogens associated with pneumonia tend to be relatively low and the population is characterized by a smaller body stature, typical of Asian populations.^34^ However, the external validity of our findings may be limited in countries or regions with higher pathogen resistance rates and populations with a large body stature.

In conclusion, the use of a ceftriaxone regimen of 2 g/day versus 1 g/day in patients with pneumonia showed no significant difference in 30-day in-hospital mortality. However, in patients with severe pneumonia requiring mechanical ventilation, the 2 g/day regimen was associated with lower 30-day in-hospital mortality. Although doses exceeding 1 g/day may not be necessary for routine pneumonia treatment, a 2 g/day regimen may be considered for patients with severe pneumonia.

## Supplementary Material

dkaf189_Supplementary_Data

## Supplementary data

Figures S1–S4 and Tables S1–S4 are available as Supplementary data at *JAC* Online.
