# Supplementary material for: Outcomes of ceftriaxone 2 g versus 1 g daily in hospitalized patients with pneumonia: a nationwide retrospective cohort study
Source: J Antimicrob Chemother. 2025 Jun 10;80(8):2194–202. doi: 10.1093/jac/dkaf189 (PMC12313466; doi:10.1093/jac/dkaf189)
Supplement: dkaf189_Supplementary_Data [file dkaf189_supplementary_data.docx]

**Supplementary Table 1**. **ICD-10 codes used to define each comorbidity**

| Hypertension | I10.x, I11.0, I11.9 |
| --- | --- |
| Diabetes mellitus | E10.x–E14.x |
| Dyslipidemia | E78.x |
| Chronic obstructive pulmonary disease | J43.x, J44.x |
| Interstitial Pneumonia | J84.x, J99.x |
| Bronchiectasis & NTM of the lungs | A31.0, J47.x |
| Fungal lung disease | B37.1, B38.0, B38.1, B38.2, B39.0, B39.1, B39.2, B40.0, B40.1, B40.2, B41.0, B42.0, B44.0, B44.1, B45.0, B46.0 |
| Lung cancer | C34.x |
| Chronic respiratory failure | J96.1 |
| Esophageal disorders and dysphagia | C15.x, F45.3, J39.8, J95.0, J98.0, K11.7, K22.0, K22.2, K22.5, K22.8, K44.9, R13.x, R19.8 |
| Cerebrovascular disease | I60.x–I69.x |
| Neurologic disease | G12.1, G12.2, G20.x, G35.x, G70.0 |
| Cardiovascular disease | I20.x-25.x, I42.x, I50.x |
| Liver disease | B16.x-19.x, K70.x–77.x |
| Gallbladder disease | C23.x, K563, K80.x, K81.x, K82.x, K851, K915, Z904 |
| Biliary tract disease | C24.x, K743, K83.x, K918 |
| Chronic kidney failure | I12.0, N18.x–19.x |
| Hematologic malignancy | C81.x-96.x, T86.0, T86.8, T86.9, Y83.8, Z94.8, Z94.9 |
| Non-hematologic malignancy | C00.x-14.x, C16.x–C22.x, C25.x–C32.x, C35.x–C80.x, C97.x, D00.x–09.x |
| Dementia | F00.x–F03.x, G30.x |

NTM, non-tuberculosis mycobacterium.

**Supplementary Table 2**. **Sensitivity analysis of outcomes between the propensity score weighted groups in patients without combination therapy**

|  | 1 g/day | 2 g/day | Risk difference | 95% confidence interval | *P* |
| --- | --- | --- | --- | --- | --- |
| **Patients did not receive combination therapy with macrolides or tetracyclines (%)**  **[2 g/day (n = 243,566) vs. 1 g/day (n = 151,080)]** |  |  |  |  |  |
| Primary outcome |  |  |  |  |  |
| 30-day in-hospital mortality | 5.1% | 4.9% | −0.1 | −0.3 to 0.1 | 0.192 |
| Secondary outcome |  |  |  |  |  |
| Overall adverse events | 1.9% | 2.1% | 0.2 | 0.0 to 0.3 | 0.011 |
| Biliary complications | 0.3% | 0.3% | 0.0 | −0.0 to 0.0 | 0.966 |
| *Clostridioides difficile* infection | 1.2% | 1.3% | 0.1 | 0.0 to 0.2 | 0.039 |
| Allergic reactions | 0.5% | 0.6% | 0.1 | 0.0 to 0.1 | 0.045 |

**Supplementary Table 3. Sensitivity analysis using unweighted univariate logistic regression models for 30-day in-hospital mortality.**

|  | Odds ratio | 95% Confidence interbal | P |
| --- | --- | --- | --- |
| Ceftriaxone dose | 0.85 | 0.83–0.88 | < 0.001 |

**Supplementary Table 4. Sensitivity analysis using unweighted multivariate logistic regression models for 30-day in-hospital mortality.**

|  | Odds ratio | 95% Confidence interval | P |
| --- | --- | --- | --- |
| Ceftriaxone dose | 0.97 | 0.93–1.01 | 0.192 |
| Age, years | 1.02 | 1.02–1.03 | < 0.001 |
| Male | 1.75 | 1.66–1.84 | < 0.001 |
| Body weight | 0.98 | 0.98–0.98 | < 0.001 |
| Smoking history |  |  |  |
| Nonsmoker |  | Reference |  |
| Current/past smoker | 0.91 | 0.86–0.96 | < 0.001 |
| GCS on admission | 0.96 | 0.95–0.97 | < 0.001 |
| Barthel index on admission |  |  |  |
| 0 | 3.17 | 2.93–3.42 | < 0.001 |
| 5–50 | 1.96 | 1.81–2.11 | < 0.001 |
| 55–95 | 1.14 | 1.04–1.24 | < 0.001 |
| 100 |  | Reference |  |
| Charlson comorbidity index | 1.17 | 1.14–1.21 | < 0.001 |
| Pneumonia severity score | 1.61 | 1.58–1.65 | < 0.001 |
| ICU admission | 0.94 | 0.79–1.12 | 0.479 |
| HCU admission | 0.99 | 0.88–1.11 | 0.854 |
| Ambulance transport | 0.98 | 0.94–1.02 | 0.336 |
| Fiscal year |  |  |  |
| 2010–2011 |  | Refenrece |  |
| 2012–2013 | 0.99 | 0.91–1.08 | 0.815 |
| 2014–2015 | 1.07 | 0.99–1.15 | 0.109 |
| 2016–2017 | 1.11 | 1.03–1.20 | 0.007 |
| 2018–2019 | 1.10 | 1.02–1.19 | 0.009 |
| 2020–2021 | 1.39 | 1.29–1.51 | < 0.001 |
| Aspiration pneumonia | 1.01 | 0.94–1.08 | 0.878 |
| Hypertension | 0.61 | 0.58–0.64 | < 0.001 |
| Diabetes mellitus | 0.88 | 0.83–0.93 | < 0.001 |
| Dyslipidemia | 0.70 | 0.64–0.77 | < 0.001 |
| Lung disease |  |  |  |
| Chronic obstructive pulmonary disease | 0.65 | 0.60–0.71 | < 0.001 |
| Interstitial Pneumonia | 2.76 | 2.51–3.03 | < 0.001 |
| Bronchiectasis & NTM of the lungs | 1.23 | 1.03–1.48 | 0.023 |
| Fungal lung disease | 2.90 | 2.08–4.02 | < 0.001 |
| Lung cancer | 2.53 | 2.27–2.82 | < 0.001 |
| Chronic respiratory failure | 1.05 | 0.93–1.18 | 0.467 |
| Esophageal disorders and dysphagia | 0.89 | 0.80–0.99 | 0.028 |
| Cerebrovascular disease | 0.79 | 0.74–0.84 | < 0.001 |
| Neurologic disease | 0.71 | 0.61–0.81 | < 0.001 |
| Cardiovascular disease | 1.03 | 0.95–1.11 | 0.493 |
| Liver disease | 0.99 | 0.87–1.12 | 0.837 |
| Gallbladder disease | 0.74 | 0.58–0.95 | 0.019 |
| Biliary tract disease | 0.90 | 0.63–1.28 | 0.548 |
| Chronic kidney failure | 1.38 | 1.28–1.49 | < 0.001 |
| Hematological malignancy | 1.62 | 1.35–1.95 | < 0.001 |
| Non-hematological malignancy | 1.19 | 1.07–1.33 | 0.002 |
| Dementia | 0.53 | 0.48–0.57 | < 0.001 |
| Nutrition within 2 d of admission day |  |  |  |
| Oral feeding | 0.54 | 0.52–0.57 | < 0.001 |
| Tube feeding | 1.19 | 1.01–1.41 | 0.036 |
| Total parenteral nutrition | 1.20 | 0.94–1.54 | 0.150 |
| Treatment within 2 days of admission |  |  |  |
| Oxygenation | 1.99 | 1.89–2.09 | < 0.001 |
| Mechanical ventilation | 2.27 | 2.04–2.53 | < 0.001 |
| Vasopressors | 1.61 | 1.44–1.80 | < 0.001 |
| Renal replacement therapy | 1.19 | 1.02–1.38 | 0.026 |
| Macrolides | 1.00 | 0.93–1.07 | 0.966 |
| Tetracyclines | 1.15 | 0.99–1.32 | 0.060 |
| Steroids | 1.19 | 1.10–1.29 | < 0.001 |
| Proton pump inhibitors | 0.98 | 0.93–1.08 | 0.976 |
| Hypnotics | 1.00 | 0.93–1.08 | 0.976 |
| Antipsychotics | 0.86 | 0.82–0.90 | 0.548 |
| Teaching hospital admission | 0.86 | 0.82–0.90 | < 0.001 |

**
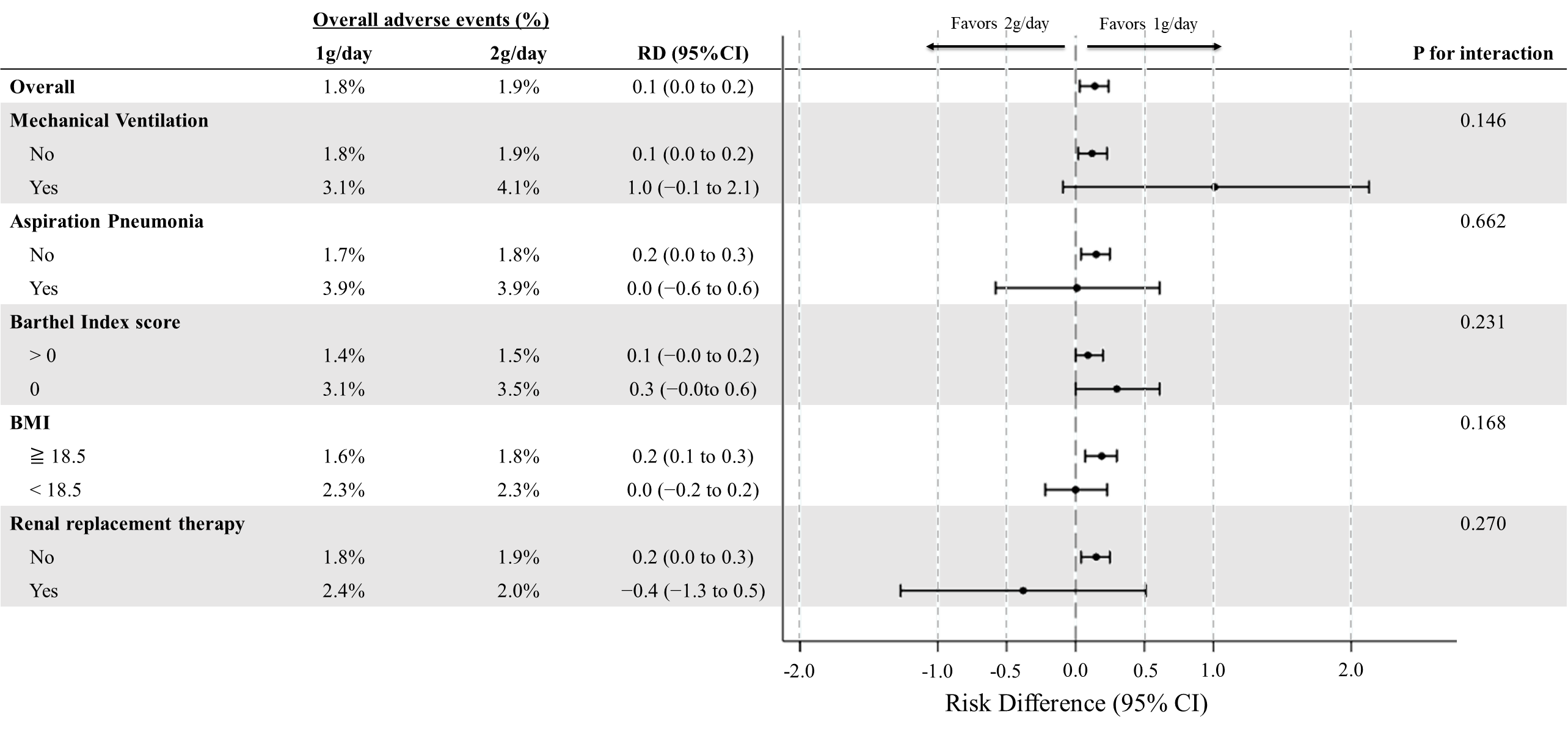
**

**Supplementary Figure 1. Subgroup analysis for overall adverse events between the propensity score weighted groups**

BMI, body mass index; RD, risk difference; CI, confidence interval.

**
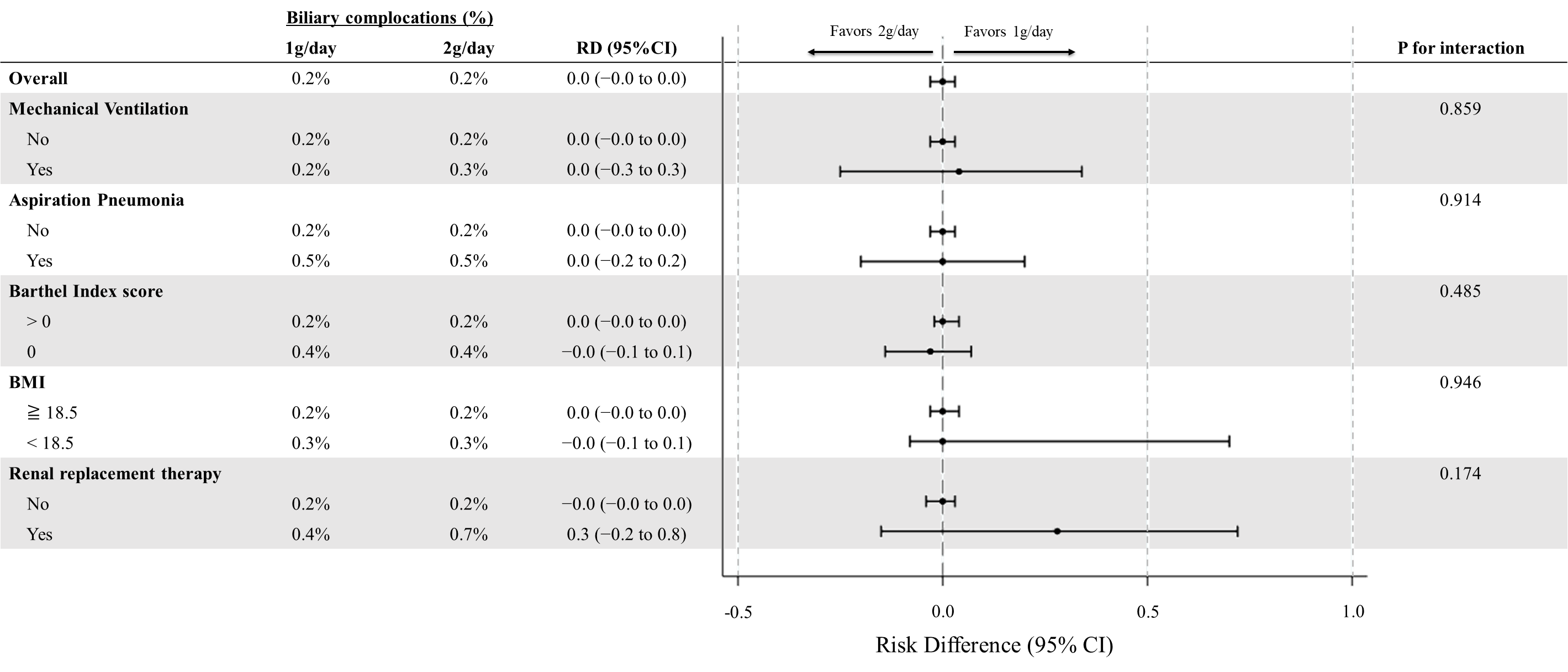
**

**Supplementary Figure 2. Subgroup analysis for biliary complications between the propensity score weighted groups**

BMI, body mass index; RD, risk difference; CI, confidence interval.

**
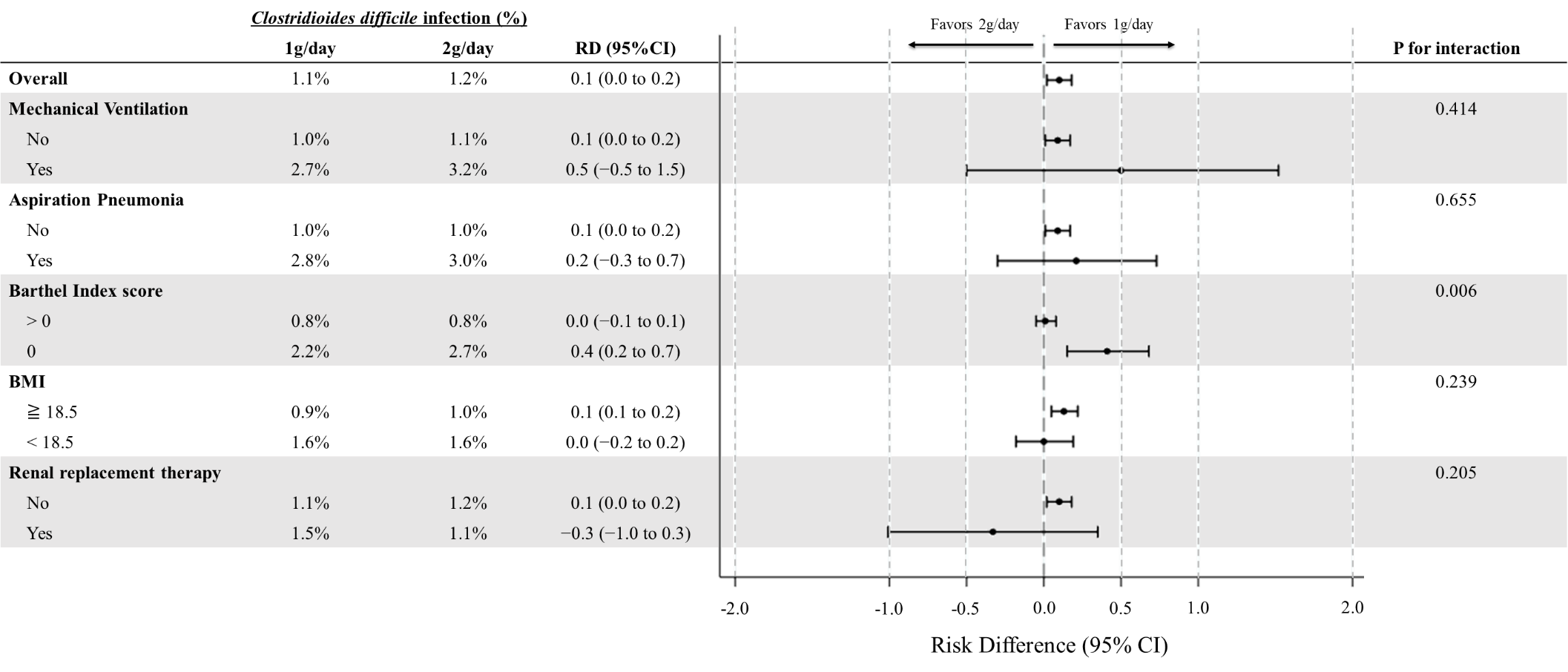
**

**Supplementary Figure 3. Subgroup analysis for *Clostridioides difficile* infection between the propensity score weighted groups**

BMI, body mass index; RD, risk difference; CI, confidence interval.

**
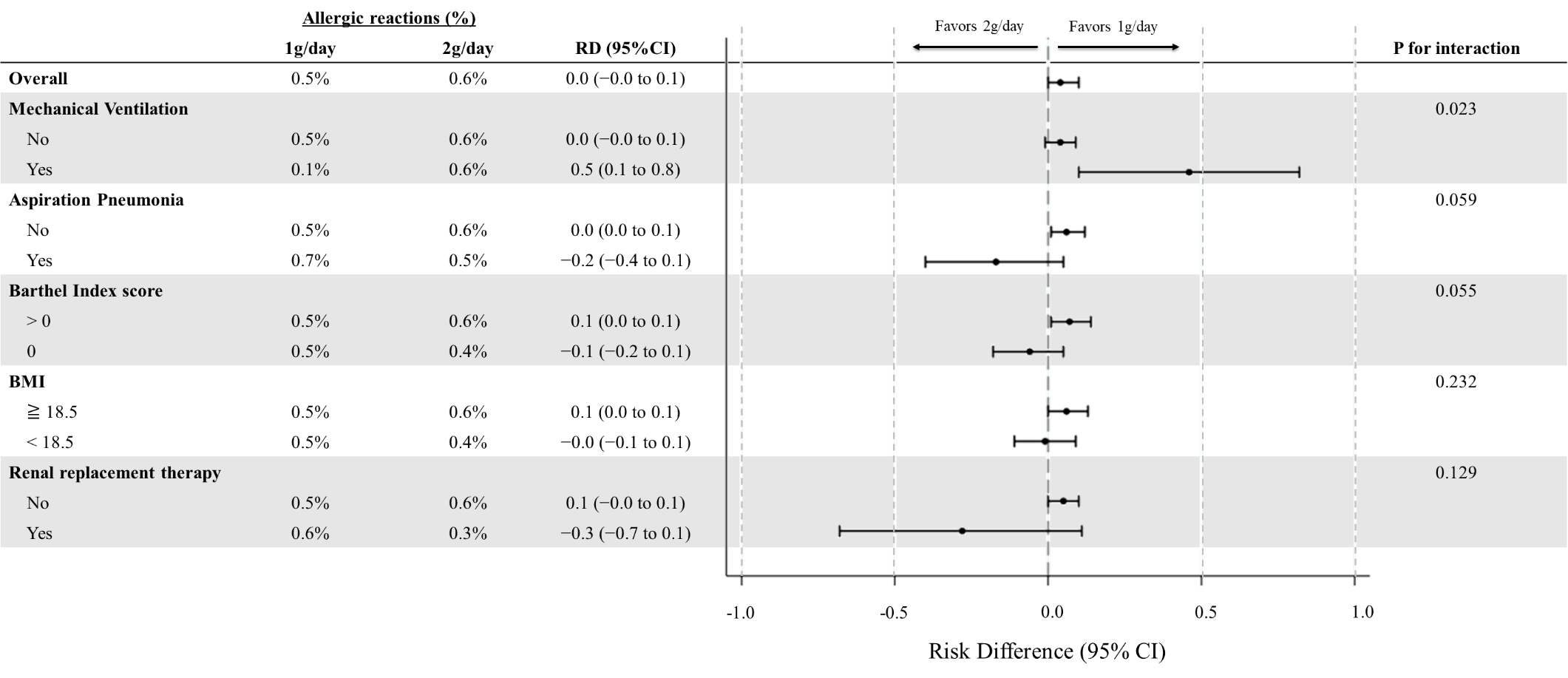
**

**Supplementary Figure 4. Subgroup analysis for allergic reactions between the propensity score weighted groups**

BMI, body mass index; RD, risk difference; CI, confidence interval.
